# Bilateral foam polidocanol sclerotherapy of great saphenous veins and their tributaries in synchronous procedure

**DOI:** 10.1590/1677-5449.200178

**Published:** 2021-06-16

**Authors:** Luiz Antonio Miranda, Rachel Cristina do Carmo, Cláudia Carvalho Sathler-Melo, Guilherme de Castro-Santos

**Affiliations:** 1 Hospital Infantil Padre Anchieta, Belo Horizonte, MG, Brasil.; 2 Hospital Militar de Área de Manaus – HMAM, Manaus, AM, Brasil.; 3 Clínica Angiovasc, Nova Lima, MG, Brasil.; 4 Universidade Federal de Minas Gerais – UFMG, Belo Horizonte, MG, Brasil

**Keywords:** foam sclerotherapy, bilateral varicose veins, complications

## Abstract

**Background:**

Chronic venous insufficiency is a highly prevalent disease. Advanced cases have high morbidity.

**Objectives:**

To evaluate the risks and benefits of foam sclerotherapy in patients who underwent bilateral treatment of the great saphenous veins in a single procedure, in selected cases of advanced venous insufficiency.

**Methods:**

We retrospectively reviewed 55 patients (110 limbs) with bilateral incompetence of the great saphenous veins who had undergone foam sclerotherapy treatment bilaterally, using a maximum dose of 20 ml of foam per patient and inelastic compression.

**Results:**

In 81 (73.6%) of the 110 saphenous veins analyzed, occlusion was obtained in the first session. After a second session this figure rose to 106 (96.3%) and all 110 (100%) veins were occluded after three sessions. Bilateral occlusion of the great saphenous veins was achieved in 27 patients (50%) in one session, in 34 (62%) patients in two sessions, and in 55 (100%) patients in three sessions. At 42 days after sclerotherapy, there was complete ulcer healing in seven (63%) of the 11 patients with ulcers and partial healing in 3 (27%) of these patients. One patient (1.8%) had self-limited lipothymia and visual scotomas, 3 patients (5.45%) had skin spots, and 19 patients (34.5%) developed retained intravascular coagulum.

**Conclusions:**

Bilateral foam sclerotherapy in a synchronous procedure is an option to be considered for treatment of varicose veins of the lower limbs.

## INTRODUCTION

Varicose veins in the lower limbs have been known since antiquity, when the Egyptians referred to them as “snakes” coiled around the legs. In the V century BC, Hippocrates likened them to “bunches of grapes” and suggested that an incandescent iron could cure them. In 1882, Tredenlegurger performed vein stripping, laying the foundations for a surgical treatment that is still used today.^1^

Until the last century, conventional surgery was the gold standard treatment for lower limb varicose veins. We evolved to minimally invasive surgery with the introduction of the crochet needle (1974), ablation with endolaser and radio frequency, and foam sclerotherapy.^2^

The first publication on foam sclerotherapy was authored by Orbach, in 1950.^3^ Cabrera, Monfreux, and Tessari et al. are responsible for the current developments.^4^^,^^5^ In 1963, Lunkeinheimer was the first to use polidocanol, which is the sclerosant agent most used in chronic venous insufficiency treatment.^6^ Polidocanol can be administrated as a sclerosant in liquid form or as foam, of which foam sclerotherapy achieves greater efficacy.^7^ Using the sclerosant in the form of microfoam, displacing the blood in the vessel, minimizes dilution and makes it easier to determine its intravenous concentration, which is not the case when it is in liquid form.^7^ It is associated with an allergic reaction in 3:1,000 administrations.^8^ In 2003, the journal Archives of Dermatology published an editorial entitled “Foam sclerotherapy: a new era”, discussing developments in ultrasonography and predicting a promising future for foam sclerotherapy, which had reached a point of no return.^9^

A bilateral approach to foam sclerotherapy consists of treating both limbs in a single session. In theory, the hypotheses justifying its use are reduced number of sessions and relief from the symptoms of chronic venous insufficiency. There are few studies in the literature that assess its risks and benefits.

The objective of this article is to describe the technique, the results, and the complications of bilateral polidocanol foam sclerotherapy of the great saphenous veins.

## MATERIALS AND METHODS

**Figure 1:**
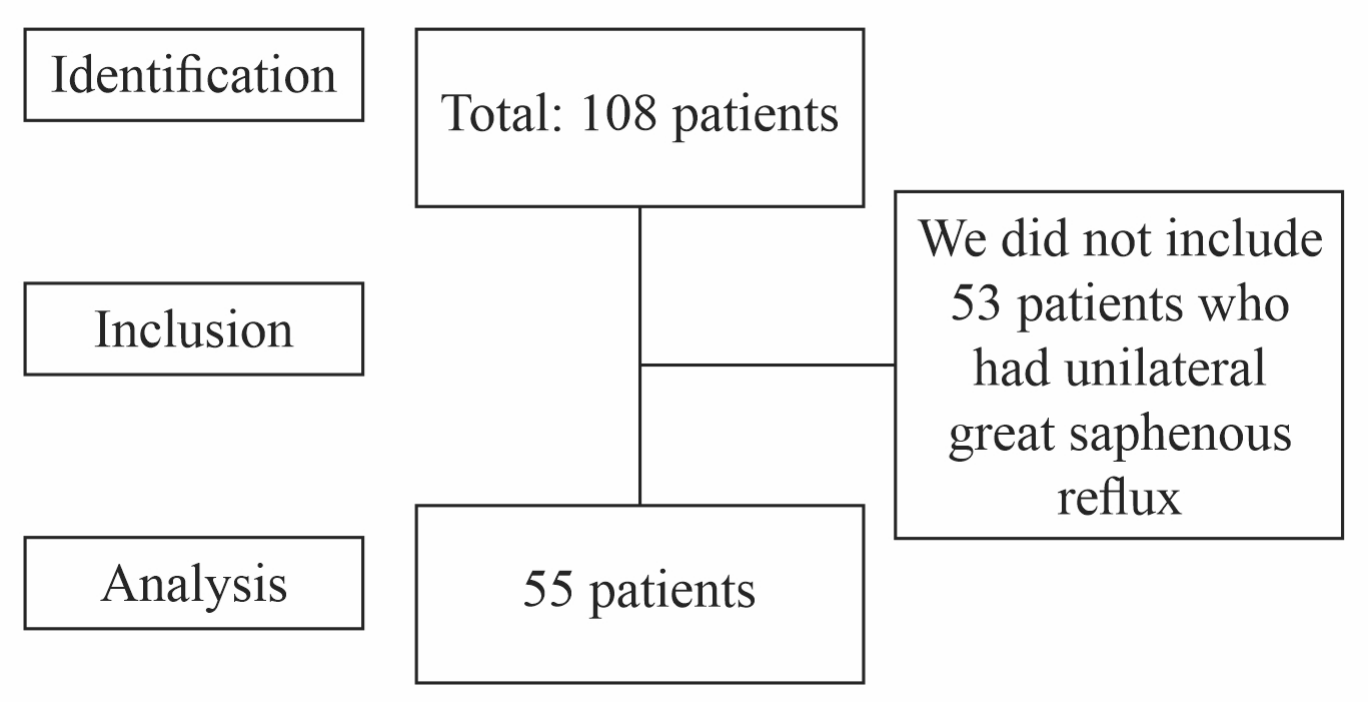
Flow diagram illustrating patient selection.

From December 2019 to March 2020, 108 patients with advanced chronic venous insufficiency were referred to us for surgical treatment and/or foam sclerotherapy on the Brazilian National Health Service (Sistema Único de Saúde) at the Hospital Infantil Padre Anchieta, in Belo Horizonte, MG, Brazil. Fifty-five of these patients who had bilateral ostial and/or segmental reflux of the great saphenous veins, had both limbs treated in a single session, and were assessed retrospectively (Figure 1). The study was evaluated and approved by the Ethics Committee (decision number 4.245.303).

**Figure 2:**
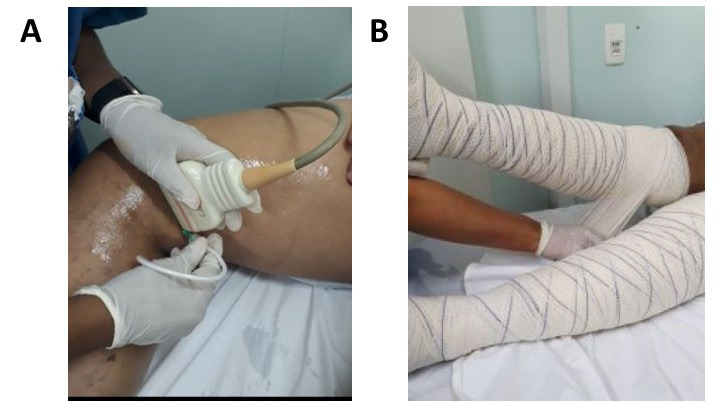
(A) Ultrasound-guided puncture and (B) compressive bandaging.

Ultrasound-guided puncture (Figure 2A) was effected using a 30/8 needle fitted to an extender, at the level of the thigh. The transducer was positioned with the right corner against the great saphenous vein, at an angle of around 45 degrees, to view penetration perpendicular to the needle. A total of 10 mL of foam was injected into each limb, 5 mL of 3% polidocanol into the great saphenous vein and 5 mL of 1% polidocanol into ipsilateral tributaries of the great saphenous vein, making a total of 20 mL per patient.

After foam injection, limb compression was applied using 15 cm crepe bandages. The bandaging technique employed was overlapping layers in a figure-of-eight pattern (Figure 2B). Patients were given the following instructions: remove bandages 24 hours after the procedure; wear elastic stockings during the day, or re-bandage the limbs; walk around and continue normal daily activities. Return visits were scheduled for 2 weeks after the procedure. At this visit, compressibility and presence or absence of flow in the varicose veins were assessed with ultrasound. Veins that were still patent were injected with foam again. Remnant coagula were diagnosed on the basis of pain and sensitivity along the venous path combined with intraluminal echogenicity on vascular ultrasound with Doppler. When coagula were found, needle puncture and percutaneous drainage were performed.

The sample size was calculated using the following formula: n = [Px(1-P)]/[SE]2,^10^ where n is sample size, P is the proportion of occurrence of the event of interest, and SE is the standard error. Based on a study by Bhogal et al.,^11^ we assumed an 81% rate of saphenous vein occlusion 2 weeks after a single session. Considering P = 0.81 and a standard error of 4%, we would need a minimum of 94 limbs.

Data were expressed as mean (± standard deviation or standard error of the mean) and counts. The Shapiro-Wilk test was used to determine normality. Non-categorical variables, such as vein diameters and mean clinical, etiological, anatomic, and pathological (CEAP) classification scores were analyzed using Student’s *t* test. Categorical variables, such as presence or absence of occlusion of saphenous veins in different subsets, were analyzed using the chi-square test with Yates’ correction or the Fischer test when appropriate. Results with p < 0.05 were considered statistically significant. All statistical analyses were conducted using Prism 8 for iOS version 8.0.1 (GraphPad Software Inc, San Diego, California).

## Results

The sample comprised a total of 55 patients, 39 of whom were female and 16 of whom were male. The youngest was 36 years old and the oldest was 68 (mean age was 50.7 years). Mean diameter of the great saphenous veins measured at the mid third of the right and left thighs was 6.2 mm (Table 1). The CEAP score for the limb with the most advanced venous insufficiency was C2 in 7 patients; C3 in 10 patients; C4 in 22 patients; C5 in 5 patients; and C6 in 11 patients (Table 2).

**Table 1:** Individual characteristics of the patients.

Age (mean, range)	50.4 (35-68)
Male sex (n%)	16 (29%)
Diameter of right great saphenous (mean ± standard deviation)	6.31±1.23
Diameter left great saphenous (mean ± standard deviation)	6.00±2.04

**Table 2:** Clinical, etiological, anatomic, and pathological classification (CEAP). C1 – telangiectasies or reticular veins; C2 – varicose veins; C3 – edema; C4 – skin changes attributable to venous disease; C5 – healed ulcer; C6 – active ulcer.

CEAP classification	n	%
C1	0	0.00
C2	7	13.00
C3	10	18.00
C4	22	40.00
C5	5	9.00
C6	11	20.00
Total	55	100.00

**Figure 3:**
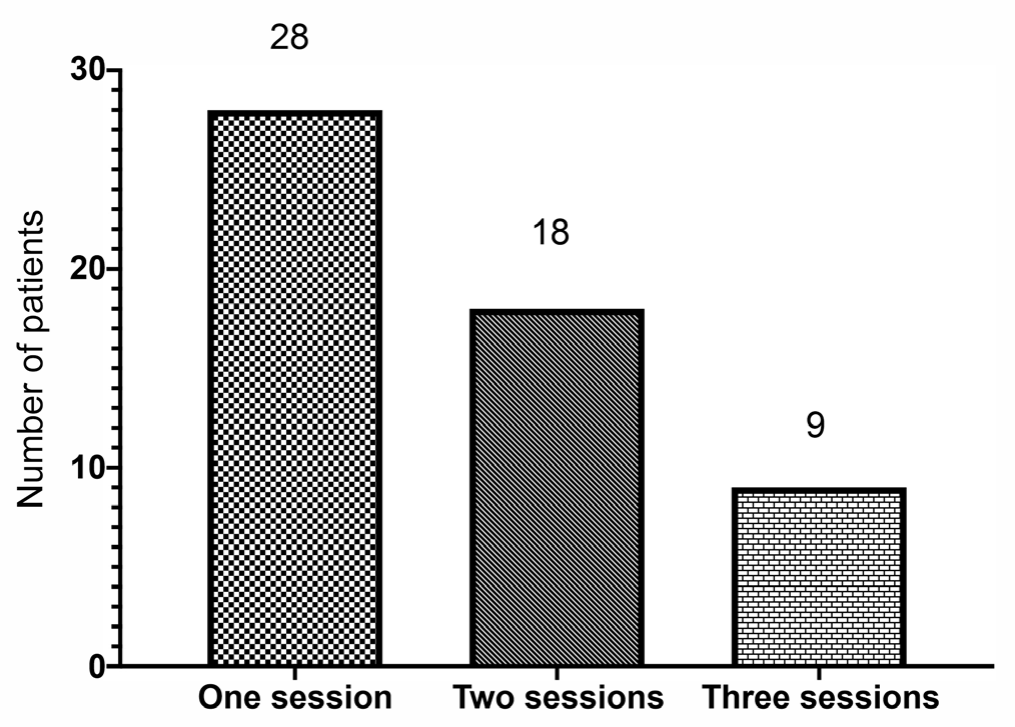
Number of patients who needed one session, two sessions, or three sessions of polidocanol foam sclerotherapy to achieve 100% bilateral occlusion of great saphenous veins.

**Figure 4:**
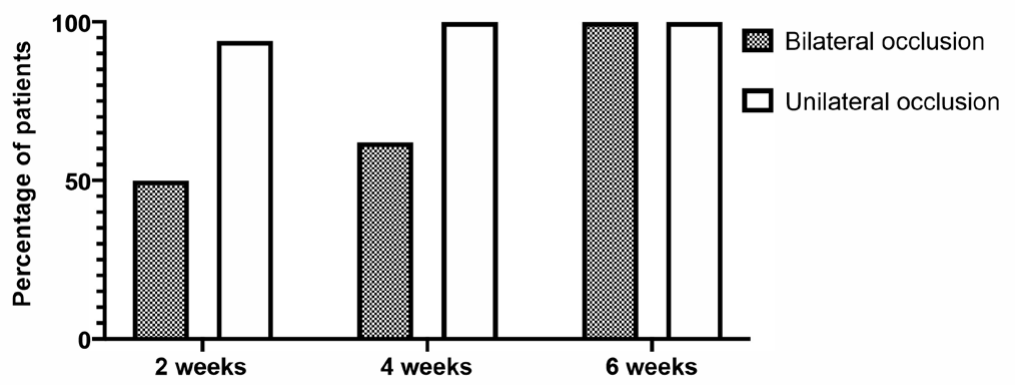
Percentage of patients with unilateral or bilateral occlusion of great saphenous veins by time

Occlusion was achieved in the first session in 81 (73.6%) of the 110 saphenous veins analyzed. After a second session this figure rose to 106 (96.3%) and all 110 (100%) veins were occluded after three sessions. Bilateral occlusion of the great saphenous veins was achieved in 27 patients (50%) in one session, in 34 (62%) patients in two sessions, and in 55 (100%) patients in three sessions (Figures 3 and 4).

No deep venous thrombosis events were observed. There was complete ulcer healing in 7 (63%) of the 11 patients with ulcers and partial healing in 3 (27%) of them, 42 days after sclerotherapy. One patient (1.8%) had self-limited lipothymia and visual scotomas, three patients (5.45%) had skin spots, and 19 patients (34.5%) developed intravascular retained coagulum.

Twelve (63%) of the patients who developed retained coagula needed puncture for drainage on one occasion and 9 (47%) needed punctures on two occasions (Table 3). Table 4 lists the CEAP classifications for subsets grouped by the number of punctures required for coagulum drainage.

**Table 3:** Number of punctures for drainage of retained coagula on separate occasions and number of patients in each subset.

Number of punctures for drainage of coagulum	Number of patients
0	36
1	12
2	7
Total	55

**Table 4:** Clinical, etiological, anatomic, and pathological classification (CEAP) in subsets grouped by need for punctures for drainage of retained coagula on separate occasions. There were no statistical differences between groups.

CEAP classification	0 punctures	%	1 puncture	%	2 punctures	%
C1	0	0.00	0	0.00	0	0.00
C2	5	14.00	2	17.00	0	0.00
C3	7	19.00	2	17.00	0	0.00
C4	16	44.00	3	25.00	3	43.00
C5	3	8.00	0	0.00	2	29.00
C6	5	14.00	5	42.00	2	29.00
Total	36	100.00	12	100.00	7	100.00

Comparison of patients in which bilateral occlusion of the saphenous veins was achieved in the first session with those in which occlusion was unilateral revealed no significant differences in CEAP classification, saphenous vein caliber, or occurrence of thrombophlebitis. There were also no statistically significant differences in venous diameters or CEAP classification between patients who needed one, two, or three sessions for occlusion of both great saphenous veins.

There were 11 patients with CEAP classification C6 and ulcers of varying degrees of severity. One patient (1.8%) was lost to follow-up at the 3-week review consultation. Seven (70%) of the remaining 10 patients’ ulcers healed. Those of the other three (30%) patients were partially healed, while ulcer size initially increased in one limb of a patient who had bilateral ulcers.

## DISCUSSION

Sclerotherapy with polidocanol foam is a rapid procedure that is safe and effective for treatment of varicose veins and chronic venous insufficiency of the lower limbs. The great majority of authors recommend its use in unilateral treatments. Patients with severe bilateral symptoms have historically been treated one limb at a time.^12^ This strategy may not be appropriate for patients who need rapid treatment, for example, those with ulcers involving both lower limbs. Sclerotherapy of great saphenous veins with polidocanol foam performed bilaterally in a single session is a technique that has been studied little.^11^

**Figure 5:**
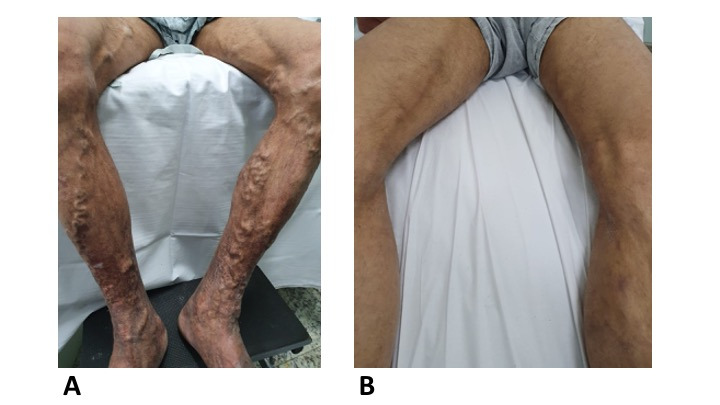
Images (A) before and (B) 2 weeks after the procedure. This patient underwent one session of sclerotherapy with 3% polidocanol foam in the great saphenous veins and 1% polidocanol foam in varicose tributaries treated bilaterally in a single session with a total volume of 20 mL. He also underwent one puncture session for drainage of retained coagulum.

Bilateral sclerotherapy of the great saphenous veins proved effective, both from the ultrasonographic point of view and from the clinical point of view (Figures 5A and 5B). In this study, 73.6% occlusion of great saphenous was observed after one session; 96.3% after two sessions, and 100% of the great saphenous were occluded after three sessions. In 50% of the patients, bilateral occlusion of the great saphenous veins was achieved in a single session. Similar results have been observed in other studies. In 2010, Bhogal et al. described 61 patients (122 limbs) treated with polidocanol foam sclerotherapy of the great saphenous veins bilaterally in a single session and 51 patients (102 limbs) treated in two sessions.^11^ They used studies by Rabe et al.^13^ and the Australian-Asian Consensus on ultrasound-guided sclerotherapy^14^ to define the volume of polidocanol foam to administer. Using a mean volume of 17.3 mL of foam for bilateral treatment in a single session and 10 mL per session in the two-session group, they achieved closure of saphenous trunks in 81% and 70% respectively. There was no increase in complications in either group in relation to the other and the higher occlusion rate was in the group treated in a single session. On this basis, these authors suggested that bilateral treatment in a single session is a safe and effective option in selected patients.^11^

Clinically, we can infer that full healing (70%), and partial healing (30%) of ulcers in C6 patients over a mean period of 42 days is indicative of improved quality of life in this study. The Venous Clinical Severity Score (VCSS) has been used to assess the severity of chronic venous insufficiency using numerical parameters, one of which is presence of ulcers.^15^^,^^16^ Similar results were found by Silva et al.,^17^ who observed 89% closure of ulcers at 791 days in 19 patients who underwent sclerotherapy with polidocanol foam. In a study by Howard et al.,^18^ an 86% ulcer healing rate was observed at 12 months. In 2013, Coelho-Neto et al. published a study on treatment of patients with an advanced predominant CEAP class, either C5 or C6. They treated unilaterally and employed a maximum dose of 10 mL per session, achieving 58% success in a single session. There was statistically significant clinical improvement in ulcer healing in 85% of the patients at 30 days.^19^

The recanalization rate is higher with foam sclerotherapy than with conventional surgery for treatment of reflux in the great saphenous veins. Figueiredo et al.^20^ conducted a pioneering randomized study that observed occlusion rates of 90% for open surgery and 78% for foam sclerotherapy at 180-day follow-up. However, even in those patients with recanalized saphenous veins, reductions were observed in venous caliber and severity of symptoms.^21^

In 2006, Wright published a randomized study conducted in Europe to compare foam sclerotherapy and surgery, observing a 5.3% incidence of deep venous thrombosis when up to 60 mL of foam was injected, which prompted the author to reduce the maximum volume to 30 mL. After this reduction, 95 patients were treated with no additional episodes of deep venous thrombosis.^21^

A unilateral approach is uncontroversial if limited to the 10 mL recommended by the European Guidelines for Sclerotherapy in Chronic Venous Disorders, since the maximum recommended foam volume is 10 mL per session in routine cases.^12^ The Australian-Asian consensus on foam sclerotherapy permits larger maximum doses.^14^ In this consensus, larger foam volumes were used on the basis of studies by authors who used volumes of around 20 mL or even larger per session. In 2019, Khan Kharl et al. published a prospective observational study conducted in Pakistan with 662 patients with 752 lower limbs affected by varicose veins, 59% of whom were C2 and C3 patients. They set the maximum dose of 3% polidocanol foam at 20 mL and used inelastic compression. All were treated unilaterally even though 13.5% had bilateral involvement. The rate of saphenous occlusion after a single session was 67.5%, rising to 93.6% after two sessions, and 99.4% after three sessions. Deep venous thrombosis occurred in three (0.45%) patients, and two (0.3%) patients underwent phlebectomy for superficial thrombophlebitis.^22^ Bhogal et al. used a median dose of 17.3 mL of foam for bilateral sclerotherapy in a single session.^11^ Wright used a maximum volume of 30 mL of foam when comparing sclerotherapy with Varisolve^®^ to surgery or conventional sclerotherapy.^23^

The benefits achieved with the bilateral approach (albeit by raising the foam dosage beyond the recommended limit) is occlusion of saphenous veins at rates and numbers of sessions similar to that achieved with unilateral treatment, thereby increasing the number of patients treated. One inconvenience is that the bilateral approach theoretically increases post-procedural discomfort. In this study, there was a high rate of retained coagula, observed in 19 (34.5%) patients. Similar results were observed by Ceratti et al.,^24^ who reported 11%, Nael and Rathbun^25^ with 17%, and Kurnicki et al.,^26^ with 21%. Tremaine et al.^27^ assessed sclerotherapy in upper limbs and observed a 61.9% rate of retained coagula. Patients who exhibited retained coagula were treated by needle puncture and drainage of the coagula. Patients who needed more that one drainage session tended to have more advanced chronic venous insufficiency.

More severe complications, such as deep venous thrombosis and permanent neurological damage, were not observed. There were no episodes of deep venous thrombosis in this follow-up. One patient (1.8%) had lipothymia. These findings are in line with the literature. Transient ischemic attacks after foam sclerotherapy were diagnosed in a series of 1,025 patients,^28^ with complete resolution of the symptoms after 30 minutes. Strokes have been described in the medical literature in three patients, all with a patent foramen ovale.^29^

All of the patients in this study had inelastic compressive bandages applied to the limbs in the immediate postoperative period. They were also instructed to wear elastic stockings in the early postoperative period. The therapeutic and prophylactic indications for elastic stockings, bandaging, and pneumatic compression are well-established. The International Compression Club approved a consensus on this evidence at a meeting held in Paris in November 2007.^30^ However, there is no definitive position on compression after sclerotherapy of the saphenous trunks. The guidelines recommend that it should be used and can be applied with elastic stockings or bandages.^26^ Hamel-Desnos conducted a randomized clinical trial of 60 patients either wearing elastic stockings or not using compression after saphenous closure and did not observe differences in the rate of occlusion of the saphenous veins, complications, or quality of life questionnaire responses.^31^

There is also a third option in addition to inelastic compression or elastic stockings with or without specific pressures, which is mixed compression. In 2017, Welsh conducted a systematic review that found better results in terms of comfort, tolerability, and quality of life with mixed compression when compared with elastic or inelastic compression separately.^32^

This study has several limitations. It is a retrospective study with a small number of patients. However, this is an issue that is still undecided and there are few publications on the subject. There is a gap left by the absence of literature comparing treatment with foam sclerotherapy of patients with bilateral chronic venous insufficiency bilaterally in a single session against treatment in separate sessions. This study provides data for clinical practice. Patients with advanced and bilateral chronic venous disease who have difficulties with access to healthcare systems could benefit from the technique described in this study. There is a need for more and larger studies, with larger patient samples and, preferably, with prospective and randomized designs.

## CONCLUSIONS

Sclerotherapy with polidocanol foam of great saphenous veins in a single session proved to be a safe and effective technique in selected patients.
