# Supplementary material for: Bilateral foam polidocanol sclerotherapy of great saphenous veins and their tributaries in synchronous procedure
Source: J Vasc Bras. 2021 Jun 16;20:e20200178. doi: 10.1590/1677-5449.200178 (PMC8218854; doi:10.1590/1677-5449.200178)
Supplement: Translation

# Translation: Escleroterapia com espuma de polidocanol em veias safenas magnas e suas tributárias bilateralmente em tempo único

**Authors:** Luiz Antonio Miranda, Rachel Cristina do Carmo, Cláudia Carvalho Sathler-Melo, Guilherme de Castro-Santos

**Affiliations:** 1 Hospital Infantil Padre Anchieta, Belo Horizonte, MG, Brasil.; 2 Hospital Militar de Área de Manaus – HMAM, Manaus, AM, Brasil.; 3 Clínica Angiovasc, Nova Lima, MG, Brasil.; 4 Universidade Federal de Minas Gerais – UFMG, Belo Horizonte, MG, Brasil

**Keywords:** escleroterapia com espuma, varizes bilaterais, complicações

## Abstract

**Contexto:**

A insuficiência venosa crônica é uma entidade com alta prevalência. Os casos avançados apresentam morbidade elevada.

**Objetivos:**

Avaliar os riscos e benefícios da escleroterapia com espuma de polidocanol em pacientes que foram submetidos ao tratamento das veias safenas magnas bilateralmente em tempo único.

**Métodos:**

Foram revistos retrospectivamente 55 pacientes (110 membros) portadores de incompetência bilateral das veias safenas magnas submetidas a tratamento escleroterápico com espuma bilateralmente, em tempo único, usando uma dose máxima de 20 mL de espuma de polidocanol por paciente.

**Resultados:**

Das 110 safenas analisadas, obteve-se a oclusão de 81 (73,6%) com uma sessão, de 106 (96,3%) com duas sessões e de 110 (100%) com três sessões. Houve oclusão bilateral das safenas magnas em 27 pacientes (50%) em uma sessão, em 34 (62%) em duas sessões e em 55 (100%) em três sessões. De 11 pacientes portadores de úlceras, houve cicatrização total de sete (63%) e parcial de três (27%) 42 dias após a escleroterapia. Houve lipotimia autolimitada e escotomas visuais em um paciente (1,8%) e manchas em três (5,45%); 19 pacientes (34,5%) foram submetidos a punção para drenagem de coágulo retido.

**Conclusões:**

A escleroterapia com espuma de polidocanol em veias safenas magnas em tempo único mostrou-se uma técnica segura e eficaz em pacientes selecionados.

## INTRODUÇÃO

As varizes dos membros inferiores são conhecidas desde a antiguidade, quando foram descritas pelos egípcios como “serpentinas” enroladas nas pernas. No século V a.C., Hipócrates as comparava com “cachos de uvas” e sugeria que ferro incandescente poderia curá-las. Em 1882, Tredenlegurger, realizando o *striping* das veias, lançou as bases para o tratamento cirúrgico que persiste até os dias atuais^1^.

Até o século passado, o padrão-ouro do tratamento das varizes dos membros inferiores era a cirurgia convencional. Evoluímos para as cirurgias minimamente invasivas com a introdução da agulha de crochê (1974), abrasão por *endolaser* e radiofrequência e escleroterapia por espuma^2^.

A primeira publicação sobre escleroterapia com espuma foi feita por Orbach, em 1950^3^. Cabrera, Monfreux, Tessari et al. são os responsáveis pelos avanços atuais^4^^,^^5^. Lunkeinheimer, em 1963, foi o primeiro a usar o polidocanol, substância esclerosante mais usada para o tratamento da insuficiência venosa crônica^6^. O polidocanol pode ser aplicado como esclerosante na forma líquida ou como espuma, sendo que a escleroterapia com espuma resultou maior eficácia^7^. O uso do esclerosante na forma de microespuma, ao deslocar o sangue no interior do vaso, minimiza a diluição do mesmo e facilita o reconhecimento de sua concentração intravenosa, o que não ocorre na forma líquida^7^. Associa-se à reação alérgica em 3:1.000 aplicações^8^. Em 2003, a revista *Archives of Dermatology* publicou um editorial intitulado “Foam sclerotherapy: a new era”, que, em conjunto com os avanços da ultrassonografia, anteviu o futuro promissor, sem volta, da escleroterapia com espuma^9^.

Uma abordagem bilateral na escleroterapia com espuma consiste em tratar os dois membros em um tempo único. Em tese, a redução do número de sessões e o alívio dos sintomas da insuficiência venosa crônica seriam hipóteses para a sua realização. Na literatura, são escassos os trabalhos avaliando seus riscos e benefícios.

O objetivo deste artigo é descrever a técnica, os resultados e as complicações da escleroterapia bilateral das veias safenas magnas com espuma de polidocanol.

## MATERIAIS E MÉTODOS

No período compreendido entre dezembro de 2019 e março de 2020, recebemos 108 pacientes portadores de insuficiência venosa crônica avançada, encaminhados para tratamento cirúrgico e/ou escleroterapia com espuma pelo Sistema Único de Saúde no Hospital Infantil Padre Anchieta, em Belo Horizonte, no estado de Minas Gerais. Desses pacientes, 55 apresentavam refluxo bilateral ostial e/ou segmentar das veias safenas magnas e tiveram os dois membros tratados em tempo único e avaliados retrospectivamente (Figura 1). Este trabalho foi avaliado e aprovado pelo Comitê de Ética (parecer número 4.245.303).

**Figura 1:** Diagrama de fluxo dos pacientes em estudo.

Foi realizada uma punção ecoguiada (Figura 2A), usando uma agulha 30/8 acoplada a um extensor, ao nível da coxa. O transdutor foi colocado com o canto direito sobre a veia safena magna e angulado a cerca de 45 graus para visualização da penetração perpendicular da agulha. Foram injetados 10 mL de espuma em cada membro, sendo 5 mL de polidocanol a 3% em veia safena magna e 5 mL de polidocanol a 1% em tributárias ipsilaterais da veia safena magna, totalizando 20 mL de espuma em cada paciente.

**Figura 2:** (A) Punção ecoguiada e (B) enfaixamento compressivo.

Para compressão dos membros, após a injeção da espuma, utilizaram-se ataduras de crepom de 15 cm. A técnica de enfaixamento foi de camadas sobrepostas em oito (Figura 2B). As orientações aos pacientes foram as seguintes: retirar as faixas 24 horas após o procedimento; utilizar as meias elásticas durante o dia ou voltar a enfaixar os membros; deambular e manter as atividades habituais. Foram agendados retornos a cada 2 semanas desde o procedimento. Nesse retorno, verificava-se a compressibilidade e a presença ou ausência de fluxo nas veias varicosas ao ultrassom. As que estavam pérvias receberam nova injeção de espuma. Os coágulos retidos foram diagnosticados considerando dolorimento e sensibilidade no trajeto venoso associado à imagem ecogênica intraluminal ao ultrassom vascular com Doppler. Em caso de coágulo retido, foi realizada punção com agulha e drenagem percutânea.

O cálculo do tamanho da amostra foi realizado conforme a fórmula: n = [Px(1-P)]/[EP]2 10, em que n é o tamanho da amostra, P é a proporção de ocorrência do evento estudado e EP, o erro padrão. Considerando o trabalho de Bhogal et al.^11^, observamos uma taxa de oclusão das veias safenas em tempo único de 81% em 2 semanas. Considerando P = 0,81 e um erro padrão de 4%, teríamos um mínimo de 94 membros.

Os dados foram apresentados como média (± desvio padrão ou erro padrão da média) e números. Foi realizado o teste de Shapiro-Wilk para avaliação da normalidade. Variáveis não categóricas, como diâmetros venoso e média da classificação clínica, etiológica, anatômica e patológica (CEAP), foram avaliadas através do teste *t* de Student. Variáveis categóricas, como presença ou não de oclusão das veias safenas em diversos grupos, foram estudadas através do teste do qui-quadrado com a correção de Yates ou do teste de Fischer quando apropriado. Um valor de p < 0,05 foi considerado estatisticamente significativo. Toda a análise estatística foi realizada através do *software* Prism 8 para iOS versão 8.0.1 (GraphPad Software Inc, San Diego, Califórnia).

## RESULTADOS

Ao total, 55 pacientes, 39 do sexo feminino e 16 do sexo masculino, fizeram parte da amostra. O mais jovem possuía 36 anos e o mais idoso possuía 68 anos (média de 50,7 anos). A média do diâmetro das safenas magnas medidas no terço médio da coxa direita e da coxa esquerda foi de 6,2 mm (Tabela 1). A CEAP do membro com insuficiência venosa mais avançada: C2, 7 pacientes; C3, 10 pacientes; C4, 22 pacientes; C5, 5 pacientes; e C6, 11 pacientes (Tabela 2).

**Tabela 1:** Características individuais dos pacientes.

Idade (média, min-máx)	50,4 (35-68)
Gênero masculino (n%)	16 (29%)
Diâmetro safena magna direita (média ± desvio padrão)	6,31±1,23
Diâmetro safena magna esquerda (média ± desvio padrão)	6,00±2,04

**Tabela 2:** Classificação clínica, etiológica, anatômica e patológica (CEAP). C1 – telangiectasias ou veias reticulares; C2 – veias varicosas; C3 – edema; C4 – alterações cutâneas atribuídas a doença venosa; C5 – úlcera cicatrizada; C6 – úlcera ativa.

Classificação CEAP	n	%
C1	0	0,00
C2	7	13,00
C3	10	18,00
C4	22	40,00
C5	5	9,00
C6	11	20,00
Total	55	100,00

Das 110 safenas analisadas, obteve-se a oclusão de 81 (73,6%) com uma sessão, de 106 (96,3%) com duas sessões e de 110 (100%) com três sessões. Houve oclusão bilateral das safenas magnas em 27 pacientes (50%) em uma sessão, em 34 (62%) em duas sessões e em 55 (100%) em três sessões (Figuras 3 e 4).

**Figura 3:** Número de pacientes que necessitaram de uma sessão, duas sessões ou três sessões de escleroterapia com espuma de polidocanol para obter 100% de oclusão bilateral das veias safenas magnas.

**Figura 4:** Porcentagem de pacientes com oclusão uni ou bilateral das veias safenas magnas em relação ao tempo

Não foi observada nenhuma ocorrência de trombose venosa profunda. De 11 pacientes portadores de úlceras, houve cicatrização total em 7 (63%) e parcial em 3 (27%) 42 dias após a escleroterapia. Houve lipotimia autolimitada e escotomas visuais em 1 paciente (1,8%) e manchas em 3 (5,45%); 19 pacientes (34,5%) desenvolveram coágulo retido.

Dos pacientes que desenvolveram coágulo retido, 12 (63%) necessitaram de punção para drenagem em uma ocasião, 9 (47%) necessitaram de punções em duas ocasiões (Tabela 3). A classificação CEAP em cada grupo a depender do número de ocasiões em que se necessitou de punção e drenagem de coágulo retido está demonstrada na Tabela 4.

**Tabela 3:** Número de punções para drenagem de coágulo retido em diferentes ocasiões e o número de pacientes em cada grupo.

Número de punções com drenagem de coágulo	Número de pacientes
0	36
1	12
2	7
Total	55

**Tabela 4:** Classificação clínica, etiológica, anatômica e patológica (CEAP) em cada grupo conforme a necessidade de punção em diferentes ocasiões para drenagem de coágulo retido. Não houve diferença estatística entre os grupos.

Classificação CEAP	0 punção	%	1 punção	%	2 punções	%
C1	0	0,00	0	0,00	0	0,00
C2	5	14,00	2	17,00	0	0,00
C3	7	19,00	2	17,00	0	0,00
C4	16	44,00	3	25,00	3	43,00
C5	3	8,00	0	0,00	2	29,00
C6	5	14,00	5	42,00	2	29,00
Total	36	100,00	12	100,00	7	100,00

Comparando-se os pacientes nos quais houve oclusão bilateral das safenas e nos quais ocorreu oclusão unilateral na primeira sessão, não houve diferença significativa quanto à classificação CEAP e calibre das safenas e quanto à ocorrência de tromboflebite. Entre os pacientes que necessitaram de uma, duas ou três sessões para oclusão das duas veias safenas magnas, não houve diferença estatisticamente significativas quanto aos diâmetros venosos e quanto à classificação CEAP.

Foram observados 11 pacientes com classificação CEAP C6, com úlceras com diferentes graus de gravidade. Na revisão de 3 semanas, houve perda de seguimento de um paciente (1,8%). Dos 10 pacientes restantes, 7 (70%) estavam cicatrizados. Os outros 3 (30%) estavam parcialmente cicatrizados, sendo que, em uma paciente que apresentava úlcera bilateral em um dos membros, houve inicialmente aumento do tamanho da úlcera.

## DISCUSSÃO

A escleroterapia com espuma de polidocanol é um procedimento rápido, seguro e eficaz no tratamento das veias varicosas e da insuficiência venosa crônica dos membros inferiores. A grande maioria dos autores defende o seu uso através do tratamento unilateral. Os pacientes que apresentam sintomas graves bilateralmente são historicamente tratados um membro por vez^12^. Essa estratégia pode não ser adequada naqueles pacientes que necessitam de rápido tratamento, por exemplo, naqueles com feridas em ambos os membros inferiores. A escleroterapia com espuma de polidocanol em veias safenas magnas realizada bilateralmente e em tempo único é uma técnica ainda pouco estudada^11^.

A escleroterapia bilateral das veias safenas magnas mostrou-se eficaz, tanto do ponto de vista ultrassonográfico como do ponto de vista clínico (Figuras 5A e 5B). Observou-se, neste estudo, oclusão de 73,6% das safenas magnas com uma sessão; de 96,3% com duas sessões e de 100% das safenas magnas em três sessões. Em 50% dos pacientes, houve oclusão bilateral das veias safenas magnas em uma única sessão. Observaram-se resultados semelhantes em outros estudos. Bhogal, em 2010, descreveu 61 pacientes (122 membros) tratados por meio de escleroterapia com espuma de polidocanol em veias safenas magnas bilateralmente em tempo único e 51 pacientes (102 membros) tratados em dois tempos^11^. O autor baseou-se nos estudos de Rabe^13^ e no Consenso Australiano-Asiático de escleroterapia venosa guiada por ultrassonografia^14^ para definir o volume de espuma de polidocanol utilizado. Usando uma média de 17,3 mL de espuma para bilaterais em tempo único e 10 mL para cada tempo no grupo em dois tempos, o autor conseguiu um fechamento dos troncos safênicos em 81% e 70%, respectivamente. Não houve aumento de complicações de um grupo em relação ao outro, com maiores taxas de oclusão do grupo tratado em tempo único. Dessa forma, os autores sugerem que o tratamento bilateral em tempo único é uma alternativa eficaz e segura em pacientes selecionados^11^.

**Figura 5:** (A) Imagem antes e (B) 2 semanas após o procedimento. Este paciente foi submetido a uma sessão de escleroterapia com espuma de polidocanol a 3% em safenas magnas e a 1% em tributárias varicosas bilateralmente em tempo único com volume total de 20 mL. Foi submetido também a uma sessão de punção e drenagem de coágulo retido.

Clinicamente, podemos inferir que a cicatrização completa (70%) e parcial (30%) das úlceras nos pacientes C6, em um tempo médio de 42 dias, indica uma melhora na qualidade de vida neste estudo. Por meio do *Venous Clinical Severity Score* (VCSS), avalia-se a gravidade da insuficiência venosa crônica através dos parâmetros numéricos, entre os quais a presença de úlceras^15^^,^^16^. Resultados semelhantes foram encontrados por Silva et al.: eles encontraram 89% de fechamento da úlcera em 791 dias em 19 pacientes submetidos a escleroterapia com espuma de polidocanol^17^. No estudo de Howard et al., foi observado um índice de cicatrização de úlcera de 86% em 12 meses^18^. Coelho-Neto et al., em 2013, publicou um estudo em tratamento de pacientes com CEAP predominante avançada C5 e C6. A abordagem foi unilateral e usou dose máxima de 10 mL por sessão. Obteve 58% de sucesso em apenas em uma sessão. Houve melhora clínica estatisticamente significativa e cicatrização das úlceras em 85% dos pacientes em 30 dias^19^.

Observa-se uma taxa de recanalização maior quando comparamos a escleroterapia com espuma à cirurgia convencional para tratamento de refluxo das veias safenas magnas. Figueiredo et al., em estudo randomizado pioneiro, encontraram uma taxa de oclusão de 90% na cirurgia aberta e de 78% na escleroterapia com espuma em 180 dias de seguimento^20^. Entretanto, mesmo nos pacientes com recanalização das veias safenas, observa-se uma redução do calibre venoso e redução da gravidade dos sintomas^21^.

Wright, em 2006, em estudo randomizado europeu comparando escleroterapia com espuma e cirurgia, reportou incidência de trombose venosa profunda em 5,3% dos pacientes ao injetar até 60 mL de espuma, o que levou o autor a reduzir o volume máximo para 30 mL. Após a redução, 95 pacientes foram tratados sem novos episódios de trombose venosa profunda^21^.

Uma abordagem unilateral desde que limitada aos 10 mL preconizados pelas *European Guidelines for Sclerotherapy in Chronic Venous Disorders* é realizada sem controvérsias, pois o volume de espuma máximo recomendado é 10 mL por sessão nos casos de rotina^12^. O Consenso Australiano-Asiático de escleroterapia com espuma permite doses máximas maiores^14^. Neste estudo, utilizou-se volumes maiores de espuma baseados em trabalhos de autores os quais usaram volumes em torno de 20 mL ou até maiores em cada sessão. Khan Kharl et al., em 2019, no Paquistão, fizeram um estudo observacional prospectivo de 662 pacientes com 752 membros inferiores afetados por varizes, sendo 59% dos pacientes C2 e C3. Esses autores fixaram a dose máxima de espuma de polidocanol a 3% em 20 mL e usaram a compressão inelástica. Embora 13,5% tivessem comprometimento bilateral, todos foram tratados unilateralmente. O índice de oclusão das safenas foi de 67,5% com uma sessão, de 93,6% com duas sessões e de 99,4 em três sessões. A trombose venosa profunda ocorreu em três (0,45%) pacientes, e dois (0,3%) pacientes foram submetidos a flebectomia por tromboflebite superficial^22^. Bhogal et al. usaram uma mediana de 17,3 mL de espuma quando foi realizada escleroterapia bilateralmente em tempo único^11^. Wright usou um volume máximo de 30 mL de espuma quando comparou a escleroterapia com Varisolve^®^ a cirurgia ou escleroterapia convencional^23^.

O benefício alcançado com a abordagem bilateral, embora elevando a dose de espuma acima da recomendada, é a oclusão das safenas em índices e sessões semelhantes aos da realizada para tratamento unilateral, aumentando o número de pacientes atendidos. Um dos inconvenientes apresentados é que a abordagem bilateral teoricamente aumenta o desconforto no pós-procedimento. Observou-se, neste estudo, uma alta taxa de coágulos retidos em 19 (34,5%) pacientes. Resultados semelhantes foram encontrados por Ceratti et al.^24^, os quais encontraram 11%, Nael e Rathbun^25^, 17% e Kurnicki et al.^26^, 21%. Tremaine et al.^27^, ao avaliarem escleroterapia em membros superiores, encontraram uma taxa de 61,9% de coágulos retidos. Os pacientes os quais apresentaram coágulo retido foram submetidos a punção com agulha e drenagem do coágulo. Os pacientes os quais necessitaram de drenagem em mais de uma ocasião tendiam a ter insuficiência venosa crônica mais avançada.

Complicações mais graves, como trombose venosa profunda e alterações neurológicas permanentes, não foram observadas. Não houve trombose venosa profunda neste seguimento. Um paciente (1,8%) apresentou lipotimia. Esses achados estão de acordo com a literatura. Ataques isquêmicos transitórios após escleroterapia com espuma foram diagnosticados em série com 1.025 pacientes^28^, com melhora completa dos sintomas após 30 minutos. Acidente vascular encefálico foi descrito na literatura médica em três pacientes, todos com forame oval pérvio^29^.

Todos os pacientes deste estudo foram submetidos a enfaixamento compressivo dos membros com faixa inelástica em pós-operatório imediato. Além disso, foram orientados a usar meias elásticas em pós-operatório precoce. As indicações terapêuticas e profiláticas para meias elásticas, ataduras e compressões pneumáticas já estão bem estabelecidas. O *International Compression Club* referendou essas evidências em reunião em Paris, em novembro de 2007^30^. Contudo, não há uma definição quanto à compressão pós-escleroterapia dos troncos safênicos. A diretriz recomenda que deve ser usada, e pode ser feita por meias elásticas ou bandagens^26^. Hamel-Desnos, em ensaio clínico randomizado em 60 pacientes com o uso de meias elásticas e sem nenhuma compressão no fechamento das safenas, não observou diferenças quanto à taxa de oclusão das veias safenas, às complicações e aos questionários de qualidade de vida^31^.

Além da compressão inelástica, das meias elásticas com pressões específicas ou não, há uma terceira opção, a compressão mista. Welsh, em 2017, em revisão sistemática, encontrou melhores resultados em termos de conforto, tolerabilidade e qualidade de vida na compressão mista quando comparada às compressões elástica e inelástica separadamente^32^.

Observam-se várias limitações neste estudo. Trata-se de um estudo retrospectivo, com número reduzido de pacientes. Entretanto, é um tema ainda em aberto, com poucas publicações. Existe um hiato quando se compara o tratamento com escleroterapia com espuma bilateralmente em tempo único ao tratamento realizado em tempos distintos em pacientes com insuficiência venosa crônica bilateral. Este estudo pode fornecer dados para a prática clínica. Pacientes com doença venosa crônica avançada e bilateral e com difícil acesso aos sistemas de saúde podem se beneficiar da técnica descrita neste estudo. Há a necessidade de maiores estudos, com maior número de pacientes e, de preferência, prospectivos e aleatórios.

## CONCLUSÃO

A escleroterapia com espuma de polidocanol em veias safenas magnas em tempo único mostrou-se técnica segura e eficaz em pacientes selecionados.
